# Chronic Thromboembolic Pulmonary Hypertension in Females: Clinical Features and Survival

**DOI:** 10.3390/jcdd9090308

**Published:** 2022-09-16

**Authors:** Yan Wu, Song Hu, Xin-Xin Yan, Fu-Hua Peng, Jiang-Shan Tan, Ting-Ting Guo, Xin Gao, Lu Hua

**Affiliations:** Center for Respiratory and Pulmonary Vascular Diseases, Department of Cardiology, National Clinical Research Center of Cardiovascular Diseases, State Key Laboratory of Cardiovascular Disease, Fuwai Hospital, National Center for Cardiovascular Diseases, Chinese Academy of Medical Sciences and Peking Union Medical College, Beijing 100037, China

**Keywords:** females, thromboembolic pulmonary hypertension, pulmonary endarterectomy, balloon pulmonary angioplasty, survival, anemia

## Abstract

Sparse data are available on the female-specific features of chronic thromboembolic pulmonary hypertension (CTEPH). We prospectively enrolled 160 consecutive female patients who were firstly diagnosed with CTEPH between 2013 and 2019 to explore their clinical phenotypes, treatment patterns, and long-term survival. The patients’ mean age was 54.7 ± 13.8 years, 70.6% provided a confirmed history of venous thromboembolism, 46 (28.8%) patients underwent pulmonary endarterectomy (PEA), 65 (40.6%) received balloon pulmonary angioplasty (BPA), and 49 (30.6%) were treated with medical therapy alone. The patients were followed for a median of 51 (34–70) months; three patients were lost to follow-up, and twenty-two patients died. The estimated survival rates at 1, 3, 5, and 7 years were 98.1% (95% CI 96.0–100), 96.9% (95% CI 94.2–99.6), 85.1% (95% CI 78.1–92.2), and 76.2% (95% CI 65.2–87.2), respectively. After adjusting for the confounders, the results of the multivariate Cox analysis showed that the presence of anemia (5.56, 95% CI 1.6–19.22) was associated with an increased risk of all-cause death, and compared with medical treatment, receiving PEA and BPA decreased the risk of death by 74% (0.26, 95% CI 0.07–0.97) and 86% (0.14, 95% CI 0.04–0.57), respectively. In conclusion, in the modern era of CTEPH treatment, invasive revascularization combined with targeted therapy display good clinical outcomes for females; anemia should be actively modified, which may lead to clinical improvements. (ClinicalTrials.gov Identifier: NCT05360992).

## 1. Introduction

Chronic thromboembolic pulmonary hypertension (CTEPH) is classified within group 4 pulmonary hypertension (PH), which is a rare but severe complication of pulmonary embolism (PE), leading to right heart failure and death. According to previous reports, the incidence of CTEPH after symptomatic PE in prospective studies ranged from 0.4% to 6.2% [1,2,3,4,5,6]. Pulmonary endarterectomy (PEA) is currently the preferred option for operable patients with marked symptomatic relief and improved long-term survival, and the 10-year survival rates were estimated to be 62–75% [7,8,9,10,11]. For inoperable patients and patients who have persistent or recurrent pulmonary hypertension (PH) after surgery, PAH targeted therapy with or without balloon pulmonary angioplasty (BPA) is the recommended treatment option [12,13,14,15].

Several subtypes of PH are characterized by female predominance, and the female sex appears to have longer survival than males after diagnosis [16,17,18]. Moreover, females and males also respond differently to medical therapy for PH [17]. However, sex-specific differences in CTEPH have been reported inconsistently in different studies. The European registry found the sex ratio was evenly balanced, and women underwent PEA less frequently than men but had better long-term survival [19]. On the other hand, 75% of CTEPH patients were females in Japan, and they showed less improvement through surgery than males [20]. Given these conflicting results, we conducted the present study to clarify the female-specific clinical features and the female long-term survival in CTEPH patients. We also examined independent predictors for all-cause death in these patients.

## 2. Methods

### 2.1. Study Cohort

Consecutively hospitalized female patients who were firstly diagnosed with CTEPH at the thrombosis center of Fuwai Hospital were entered into a database between May 2013 and May 2019. The patients’ clinical characteristics, functional and hemodynamic data, and comorbidities at diagnosis were collected during a routine diagnostic workup. The treatment regimens including PEA, BPA, and/or medical therapy were decided by a multidisciplinary team including PAH specialists, surgeons experienced with PEA, BPA interventionists, and radiologists. PEA was mainly considered for patients with: (1) New York Heart Association functional class III or IV symptoms; (2) a preoperative pulmonary vascular resistance (PVR) > 3.75 Wood units; (3) with surgically accessible stenosis and occlusion of pulmonary artery (in the main, lobar, or segmental pulmonary arteries); and (4) no severe comorbidities [21,22]. BPA was considered for patients with distal stenosis and occlusion, which were technically inoperable, or who carried an unfavorable risk/benefit ratio for PEA. The patients who refused or were technically inapplicable for PEA and BPA received targeted drugs [7,23]. PAH targeted drugs, including guanylate stimulator-riociguat, phosphodiesterase type 5 inhibitors, endothelin receptor antagonists, and prostacyclin analogue, were administrated alone or in combination according to the clinical judgement. Anemia was defined as hemoglobin less than 12 g/dL according to the World Health Organization classification.

Patients were followed-up by outpatient visit, hospitalization, phone calls, or online interviews annually, and the data cutoff was 22 February 2021. The primary outcome of the study was the time from diagnosis to the all-cause death.

### 2.2. CTEPH Diagnosis

CTEPH diagnosis was based on the following criteria [24,25]:

1. PH confirmed by right heart catheterization (mean pulmonary arterial pressure (mPAP) > 25 mm Hg, pulmonary vascular resistance (PVR) > 3 Wood units, and pulmonary arterial wedge pressure (PAWP) < 15 mm Hg at rest);
2. Mismatch on ventilation/perfusion scintigraphy or evidence of pulmonary vascular lesions on multidetector computed tomography angiography or pulmonary angiography [16,17];
3. At least 3 months of effective anticoagulation;
4. Other factors that may lead to pulmonary vascular lesions, such as arteritis, Takayasu’s disease, pulmonary artery sarcoma, fibrosing mediastinitis, pulmonary sarcoidosis, or schistosomiasis were excluded.

### 2.3. Statistical Analysis

Continuous variables were presented as mean ± SD or median (interquartile interval) as appropriate. The T test and the Mann–Whitney U test were performed to compare the difference between variables with normal and abnormal distribution, respectively. Categorical variables were described as frequencies (percentage, %), and the differences were tested by the chi-square test or Fisher’s exact test.

The Kaplan–Meier method was used to estimate the survival of female CTEPH patients. The Cox proportional hazards model was applied to identify the factors associated with death during follow-up. A multivariate Cox proportional hazards model was established based on the predictors associated with the hazard of death identified in the univariable analysis. For the variables that were associated with survival and differed significantly among the three treatment groups, sensitive analysis was performed to validate the results from the multivariate Cox proportional hazards model. A two-tailed *p* value < 0.05 was considered statistically significant. Statistical analyses were performed by using SAS (version 9.4, SAS institute, Cary, NC, USA)

## 3. Results

### 3.1. Clinical Features

During the study period, we enrolled 160 female patients who were firstly diagnosed with CTEPH in our center. The mean age was 54.7 ± 13.8 years, The median time from symptoms to diagnosis was 24.4 (9.1–60.78) months, 70.6% of patients provided a confirmed history of VTE, and 67.5% of the patients had WHO FC III/IV at diagnosis. The six-minute walking distance (6MWD) was 377 (342–442) meters, and the arterial oxygen saturation (SaO_2_) reduced to 89% [86–93%]. In hemodynamics, the mPAP was 51 ± 13 mmHg, the PVR was 9.68 (6.81–12.66) Wood units, and the mixed venous oxygen saturation (S_VO2_) was 61% (57–66%). Eight women (5%) were diagnosed with thrombophilia, twelve (7.5%) were also had antiphospholipid syndrome, and three suffered from cancer. In the overall cohort, 152 (95%) patients received targeted PAH therapy, 46 (28.8%) patients underwent PEA, 65 (40.6%) received BPA, and 49 (30.6%) were treated with medical therapy alone (Table 1). All the women received anticoagulants, with 65.54% with novel NOAC. Ninety-five percent of the patients were treated with PAH targeted therapy, 33.8% with monotherapy, and 61.3% with combination therapy.

Patients were followed for a median of 51 (34–70) months, three patients were lost to follow-up, and 22 patients died during the follow-up period. Comparing patients who survived with those who did not survive, the age at diagnosis, history of venous thromboembolism (VTE), WHO function class (FC) at diagnosis, mPAP, cardiac output (CO), PVR, SaO_2_, anticoagulants used, and comorbidities including thrombophilia, antiphospholipid syndrome and cancer were all similar; however, the S_VO2_ and main treatment modality differed. For patients who survived, 42 (30.4%), 61 (44.2%), and 35 (25.4%) were treated with PEA, BPA, and medical therapy alone, respectively. In contrast, for patients who did not survive, the rates were 18.2%, 18.2%, and 63.6%, respectively (*p* < 0.001). Moreover, the patients who survived had a lower occurrence of anemia than those died (6.5% and 22.7%, *p* = 0.027).

Table S1 provides the comparison between female patients and male CTEPH patients (n = 190) who were diagnosed during the same time period in our center. Females seemed to display more severe disease than males at diagnosis, presented with a higher WHO functional class, a lower 6MWD, a higher PVR, and reduced CO, Svo_2_, and SaO_2_. More male patients were recorded as having a history of VTE (82.6%), thrombophilia (12.1%), and coronary artery disease (9%). Regarding the main treatment modality, women underwent PEA less and BPA more than men. There were a total of 22 (13.8%) deaths in women and 23 (12.1%) in men recorded over the long-term follow-up.

### 3.2. Long-Term Survival and Risk Factors for All-Cause Death

**Figure 1:**
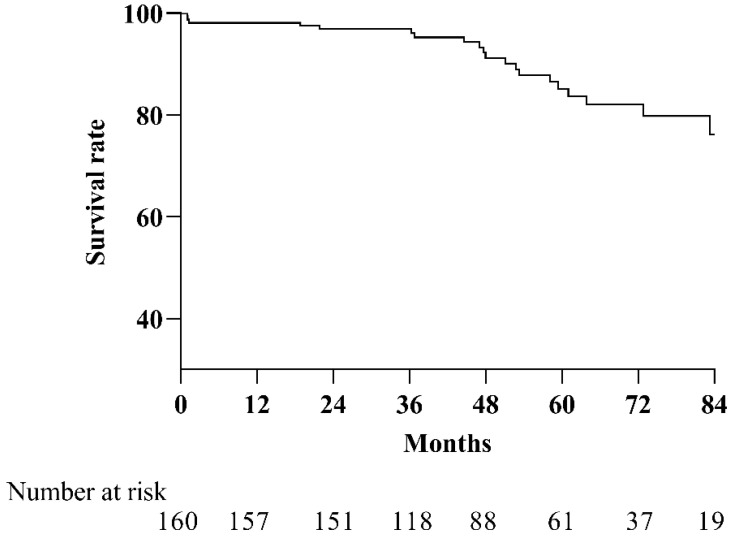
Kaplan–Meier survival for females with thromboembolic pulmonary hypertension. The estimated survival rates at 1, 3, 5, and 7 years for female patients were 98.1% (95% CI 96.0–100), 96.9% (95% CI 94.2–99.6), 85.1% (95% CI 78.1–92.2), and 76.2% (95% CI 65.2–87.2), respectively.

The estimated survival rates at 1, 3, 5, and 7 years for female patients were 98.1% (95% CI 96.0–100), 96.9% (95% CI 94.2–99.6), 85.1% (95% CI 78.1–92.2), and 76.2% (95% CI 65.2–87.2), respectively (see Figure 1). The causes of death were right heart failure (n = 15), sudden cardiac death (n = 1), perioperative death (PEA, n = 2; BPA, n = 2), and cancer (n = 2).

In the univariate COX proportional hazards regression associated with all-cause death (Table S2), the predictors with significance were retained. The results of the multivariate Cox analysis after adjusting for the confounders (age, BMI, and the predictors associated with death identified in the univariable analysis) are shown in Table 2. The presence of anemia (5.56, 95% CI 1.61–19.22) was associated with an increased risk of all-cause death, and compared with medical treatment, receiving PEA and BPA decreased the risk of death by 74% (0.26, 95% CI 0.07–0.97) and 86% (0.14, 95% CI 0.04–0.57), respectively.

### 3.3. Sensitive Analysis

The baseline clinical characteristics of patients in each treatment group (targeted drug group, PEA and BPA group) are displayed in Table S3. The WHO FC, 6MWD, mPAP, and PVR were all comparable. For the variables that differed significantly among the three groups, time from first symptom to diagnosis and anticoagulant therapy had potential clinical importance. Sensitive analysis including the two variables showed that predictors associated with death were consistent with the above results from the multivariate Cox analysis (Table S4).

### 3.4. Discussion

The present longitudinal study provided us information about the clinical phenotypes and long-term survival of female patients with CTEPH, as well as factors that were significantly associated with all-cause death. We found that patients displayed a severely impaired functional and hemodynamic status when diagnosed, and those who were still alive at the last follow-up underwent more surgical or interventional regimens than those who did not survive; the latter were mainly treated with medical therapy alone. Compared to the males diagnosed during the same time period, the females were older, had more severe disease, and underwent PEA less but BPA more than the men. Women had an estimated 5-year survival rate of 85%, and aside from the main treatment modality, anemia was determined as the most potent independent factor for all-cause mortality.

The gender distribution in our study for CTEPH was nearly balanced, which is similar to what was reported in the European registry and some other registry studies [8,19,26], but it disagreed with cohorts from the United Kingdom, Japan, Korea, and Mexico in which female predominance was observed [20,27,28,29]. The paradoxical figures may be explained by the different eligible criteria of each study and national diverse baseline features and demographics. Since CTEPH is proposed to be related to the incomplete resolution of prior VTE events, the reports from Marshall et al. that VTE-related outcomes did not appear to differ significantly based on sex may be indirect evidence for our findings [30].

In this study, we reported that the survival rates after 1, 3, 5, and 7 years for the female patients were 98.1%, 96.9%, 85.1%, and 76.2%, similar to the survival of the whole CTEPH cohort in our center, as previously reported (the survival was 97.1%, 93.3%, 86.9%, and 82.0%, respectively) [23]. When compared to the historical data from Spain and a multicenter registry study from China, the long-term survival of our female patients at 3 and 5 years was much better (Spanish: 75% and 65%, China: 84.6% and 73.4%) [31,32]. The improved survival is mainly attributed to the new therapies (PEA or BPA) developed and the technique improvement in the last ten years in our center, which has a large volume of CTEPH patients, and perhaps partly due to the female-specific characteristics. In the surviving women, nearly 75% underwent surgery or interventional therapy; in contrast, only about 36% of the non-surviving patients were treated with PEA or BPA, although all of them were treated with targeted PAH therapy. This finding is also consistent with observations from the whole CTEPH cohort in that those who received PEA or BPA had a better prognosis than those who were only treated with targeted drugs alone [23]. In the European CTEPH registry study, Barco et al. reported that women underwent PEA less often than men; of the 339 women, 183 (54%) underwent PEA, compared with 221 of the 340 men (65%) [19]. In our study, females underwent PEA less but BPA more than males; however, the invasive revascularization ratio (including PEA and BPA) was similar (69.4% and 70%). The sex-specific discrepancy in regimens for CTEPH might be explained by subjective disease-related factors that determine surgery or intervention, along with female patients’ refusal to more invasive PEA due to their older age at diagnosis.

In the multivariate analysis, we determined that receiving PEA (HR 0.26) or BPA (HR 0.14) could significantly decrease the risk of all-cause mortality compared to targeted therapy alone, and the finding was robust in the sensitivity analysis. This agreed with findings from previous studies that interventional revascularization therapy with PEA [8,31] or BPA [33] could benefit long-term survival. Moreover, we found that anemia (HR 5.56) at diagnosis was an independent factor that could significantly increase all-cause mortality in female CTEPH. Numerous mechanistic links between PH and anemia have been proposed, such as hypoxic stress, endothelial dysfunction, and inflammation-driven processes [34]. Causes of anemia in women may include erythropoiesis disorder by low cardiac output [35], iron deficiency [36], malnutrition, menorrhagia, bleeding due to anticoagulants use, and the adverse effects of endothelium receptor antagonists (bosentan, ambrisentan, macitentan) [37,38,39]. Richard et al. reported that in a cohort of 145 patients with PH (75.2% were female and 69.7% were group 1 PH), after adjustment for known predictors of death and PH etiology, anemic patients were 3.3 times more likely to die than non-anemic patients; however, in that study, only 2.8% of patients were classified in group 4 PH [40]. Although anemia has been reported to be a strong risk factor for mortality in a number of chronic illnesses [41,42], our study reveals for the first time that anemia has a powerful association with the clinical outcome in female CTEPH patients. As life-long anticoagulation is the cornerstone therapy for CTEPH, the presence of anemia may restrict the dosage and duration of anticoagulant use, even affecting the treatment choice for PEA or BPA; this may be an explanation for anemia as a marker for a worsened clinical outcome in patients. In response to hypoxic stress and chronic inflammation, anemia can be a consequence of PH. On the other hand, the chronic hypoxic condition caused by various subtypes of anemia may further aggravate the pulmonary vascular remodeling in PH [34,43]. Therefore, our finding emphasizes the importance of actively modifying anemia for female patients with CTEPH, which may lead to clinical improvement in this challenging disorder.

This study had several limitations. Firstly, regarding the study design, the database was prospectively collected, but it was retrospectively analyzed; thus, we only analyzed patients’ baseline data. Secondly, the study was performed at one single large medical center and was therefore subject to referral bias. However, 160 female CTEPH patients were enrolled in the study and were followed for a median of 51 months, and only three patients were lost to follow-up during the study period. From the qualified study, we clarified the clinical phenotype, treatment patterns, and survival rates at a large-volume center in the contemporary treatment era. Thirdly, the day of diagnosis of CTEPH was used as the starting point to determine the length of survival, but there was a time lapse after diagnosis to the initiation of BPA or PEA, generally 3 to 6 months. However, the study was from real clinical practice, hence, the time lapse was almost unavoidable.

## 4. Conclusions

In this study specifically of female patients with CTEPH, we found that patients presented with severely compromised hemodynamics at diagnosis. Nearly 70% of women received invasive revascularization treatment including PEA or BPA. The survival rates at 1, 3, 5, and 7 years were estimated to be 98.1%, 96.9%, 85.1%, and 76.2%, respectively. Anemia at diagnosis and PEA and BPA regimens were determined as independent potent predictors for long-term clinical outcome. These findings provide new insights into this specific population with the challenging disorder. In the modern era of CTEPH treatment, invasive revascularization combined with targeted therapy display good clinical outcome for females; anemia should be detected early and actively modified, which may lead to clinical improvements.

## Figures and Tables

**Table 1:** The clinical characteristics of female CTEPH patients.

Variables	All Female Patients (n = 160)	Survival (n = 138)	Non-Survival (n = 22)	*p* Value
**Characteristics**				
Age	54.7 ± 13.8	54.6 ± 13.8	55.7 ± 13.8	0.716
BMI (kg/m^2^)	23.6 ± 3.3	23.7 ± 3.2	22.9 ± 3.6	0.269
History of VTE	113 (70.6)	101 (73.2)	12 (54.6)	0.075
Time from first symptom to diagnosis (months)	24.4 [9.1, 60.78]	24.3 [9.1, 58.8]	35.0 [9.6, 66.7]	0.321
WHO FC III/IV	108 (67.5)	92 (66.7)	16 (72.7)	0.573
6MWD (m)	377 [342, 442]	384 [342, 442]	342.0 [253, 442]	0.037
**Hemodynamics**				
Systolic PAP (mmHg)	84.7 ± 23.6	84.2 ± 24.0	87.5 ± 21.2	0.542
Diastolic PAP (mmHg)	31.0 [25.5, 36.5]	31.0 [25.0, 36.0]	31.0 [26.0, 39.0]	0.661
mean PAP (mmHg)	50.6 ± 13.0	50.2 ± 13.2	53.0 ± 11.6	0.361
mean RAP (mmHg)	7.0 [4.0, 10.0]	6.0 [4.0, 10.0]	7.5 [5.0, 11.0]	0.141
PAWP (mmHg)	10.0 [8.0, 12.0]	10.0 [7.0, 11.0]	10.0 [9.0, 12.0]	0.119
PVR (Wood units)	9.68 [6.81, 12.66]	9.40 [6.79, 12.35]	11.70 [6.89, 15.00]	0.136
CO (L/min)	4.32 [3.69, 4.96]	4.34 [3.70, 5.03]	4.01 [3.00, 4.90]	0.135
CI (L/(min.m^2^)	2.47 [2.14, 2.90]	2.47 [2.18, 2.91]	2.39 [1.80, 2.72]	0.161
Svo_2_ (%)	61.1 [56.9, 65.9]	61.6 [58.1, 66.2]	56.9 [43.3, 65.5]	0.022
**Laboratory tests**				
HGB (g/L)	143.0 [129.0, 155.0]	144.5 [131.0, 158.0]	129.5 [114.0, 146.0]	0.013
PLT (×10^9^)	199.0 [162.0, 258.5]	199.5 [162.0, 263.0]	194.5 [167.0, 234.0]	0.431
NT-proBNP (ng/mL)	1318 [350, 2867]	1208 [310, 2782]	2109 [765, 4312]	0.095
D-dimer (μg/mL)	0.42 [0.22, 0.82]	0.42 [0.23, 0.77]	0.45 [0.22, 1.20]	0.338
ALT (IU/L)	20.0 [13.0, 35.0]	21.0 [14.0, 35.0]	17.0 [12.0, 35.0]	0.715
AST (IU/L)	24.0 [19.0, 31.0]	24.0 [20.0, 31.0]	22.0 [19.0, 31.0]	0.625
TB (μmol/L)	18.07 [12.90, 27.57]	18.14 [12.67, 26.30]	18.00 [15.90, 32.60]	0.455
DB (μmol/L)	3.90 [2.87, 7.40]	3.90 [2.87, 7.40]	4.30 [2.90, 7.40]	>0.999
Creatinine (μmol/L)	70.15 [61.00, 80.07]	70.75 [61.60, 80.07]	68.50 [57.06, 79.20]	0.347
Uric acid (μmol/L)	381.4 [301.1, 465.5]	386.8 [314.2, 460.3]	335.3 [284.0, 554.0]	0.690
Sao_2_ (%)	89.4 [86.6, 92.5]	89.4 [86.8, 92.5]	89.6 [84.5, 92.1]	0.818
**Comorbidities**				
Thrombophilia	8 (5.0)	7 (5.1)	1 (4.6)	>0.999
APS	12 (7.5)	11 (8.0)	1 (4.6)	>0.999
CHD	6 (3.8)	4 (2.9)	2 (9.1)	0.192
DM	4 (2.5)	3 (2.2)	1 (4.6)	0.450
Hypertension	37 (23.1)	33 (23.9)	4 (18.2)	0.554
Cancer	3 (1.9)	2 (1.5)	1 (4.6)	0.360
Anemia	14 (8.8)	9 (6.5)	5 (22.7)	0.027
**Targeted drug at diagnosis**				0.287
No drugs	8 (5.0)	8 (5.8)	0 (0.0)	
Monotherapy	54 (33.8)	44 (31.9)	10 (45.5)	
Combination therapy	98 (61.3)	86 (62.3)	12 (54.5)	
**Main treatment modality**				0.001
Targeted drug	49 (30.6)	35 (25.4)	14 (63.6)	
PEA	46 (28.8)	42 (30.4)	4 (18.2)	
BPA	65 (40.6)	61 (44.2)	4 (18.2)	
**Anticoagulants**				0.897
Warfarin	55 (34.6)	48 (34.8)	7 (33.3)	
NOAC	104 (65.4)	90 (65.2)	14 (66.7)	

Data are presented as mean ± SD or n (%) or median [IQR].CTEPH: chronic thromboembolic pulmonary hypertension; BMI: Body mass index; VTE: Venous Thrombus Embolism; WHO FC: WHO function class; 6MWD: 6-min walking distance; PAP: pulmonary artery pressure; RAP: right atrial pressure; PAWP: pulmonary artery wedge pressure; PVR: pulmonary vascular resistance; CO: cardiac output; CI: cardiac index; SvO_2_: mixed venous oxygen saturation; HGB: hemoglobin; PLT: platelet; NT-proBNP: N-terminal pro brain natriuretic peptide; ALT: alanine aminotransferase; AST: aspartic transaminase; TB: total bilirubin; DB: direct bilirubin; Sao_2_: arterial oxygen saturation; APS: antiphospholipid syndrome; CHD: coronary heart disease; DM: Diabetes mellitus; PEA: pulmonary endarterectomy; BPA: balloon pulmonary angioplasty; NOAC: novel oral anticoagulants.

**Table 2:** Results of the multivariate Cox analysis after adjusting for the confounders.

Variables	HR	95% CI	*p* Value
Age	1.01	0.97–1.05	0.741
BMI (kg/m^2^)	0.92	0.78–1.09	0.339
6MWD/20 (m)	0.97	0.90–1.05	0.415
Svo_2_ (%)	0.97	0.92–1.03	0.288
NT-proBNP/500 (ng/mL)	1.09	1.00–1.18	0.053
Cancer	5.17	0.55–48.43	0.150
Anemia	5.56	1.61–19.22	0.007
Targeted drug	Ref	Ref	
PEA	0.26	0.07–0.97	0.044
BPA	0.14	0.04–0.57	0.006

BMI: Body mass index; 6MWD/20: 6-min walking distance, per 20 m increase in distance; SvO_2_: mixed venous oxygen saturation; NT-proBNP/500: N-terminal pro-brain natriuretic peptide, per 500 ng/mL increase in concentration; PEA: pulmonary endarterectomy; BPA: balloon pulmonary angioplasty.

## Data Availability

The data presented in this study are available on request from the corresponding author. The data are not publicly available due to patients’ privacy.

## Supplementary Materials

The following supporting information can be downloaded at: https://www.mdpi.com/article/10.3390/jcdd9090308/s1, Table S1: Clinical characteristics and outcomes in female vs. male CTEPH patients; Table S2: Univariate COX proportional hazards regression analysis of risk factors for mortality in female patients; Table S3: Clinical characteristics of female CTEPH patients in each treatment group; Table S4: Sensitivity analysis results.
