# Supplementary material for: Chronic Thromboembolic Pulmonary Hypertension in Females: Clinical Features and Survival
Source: J Cardiovasc Dev Dis. 2022 Sep 16;9(9):308. doi: 10.3390/jcdd9090308 (PMC9506206; doi:10.3390/jcdd9090308)
Supplement: Supplementary file 1 [file jcdd-09-00308-s001.zip › jcdd-1810370-supplementary.pdf]

Table S1. Clinical characteristics and outcomes in female vs male CTEPH Patients.

Variables	Female (n=160)	Male (n=190)	*P* value
**Characteristics**			
Age	54.7±13.8	50.7±15.4	0.012
BMI (kg/m^2)	23.6±3.3	24.1±3.7	0.178
Smoking	2 (1.3)	101 (53.2)	<0.001
Alcohol Drinking	0 (0.0)	60 (31.6)	<0.001
History of VTE	113 (70.6)	157 (82.6)	0.008
Time from first symptom to diagnosis (months)	24.4 [9.1,60.78]	24.3 [7.5,48.7]	0.449
WHO FC III/IV	108 (67.5)	105 (55.3)	0.020
6MWD (m)	377 [342,442]	406.5 [342,453]	0.053
**Hemodynamics**			
Systolic PAP (mmHg)	84.7±23.6	82.9±21.2	0.446
Diastolic PAP (mmHg)	31.0 [25.5,36.5]	31.0 [25.0,37.0]	0.961
mean PAP (mmHg)	50.6±13.0	49.3±11.9	0.315
mean RAP (mmHg)	7.0 [4.0,10.0]	8.0 [6.0,11.0]	0.007
PAWP (mmHg)	10.0 [8.0,12.0]	10.0 [8.0,12.0]	0.116
PVR (Wood units)	9.68 [6.81,12.66]	8.07 [5.93,11.11]	0.004
CO (L/min)	4.32 [3.69,4.96]	4.66 [3.90,5.40]	0.005
CI (L/(min.m^2)	2.47 [2.14,2.90]	2.47 [2.08,2.80]	0.516
Svo_2 (%)	61.1 [56.9,65.9]	63.5 [59.6,68.5]	0.002
**Laboratory tests**			
HGB (g/L)	143.0 [129.0,155.0]	155.0 [144.0,166.0]	<0.001
WBC ($\times 10^9$)	5.71 [4.81,6.97]	6.33 [5.10,7.54]	0.005
PLT ($\times 10^9$)	199.0 [162.0,258.5]	182.0 [146.0,223.0]	0.001
NT-proBNP (ng/ml)	1318.75 [350.00,2867.65]	1129.50 [362.00,2824.00]	0.802
D-dimer (μg/ml)	0.42 [0.22,0.82]	0.58 [0.23,1.14]	0.018
ALT (IU/L)	20.0 [13.0,35.0]	25.0 [18.0,35.0]	0.001
AST (IU/L)	24.0 [19.0,31.0]	25.0 [20.0,32.0]	0.493
TB (μmol/L)	18.07 [12.90,27.57]	21.50 [15.64,32.70]	0.003
DB (μmol/L)	3.90 [2.87,7.40]	4.85 [3.20,7.95]	0.029
Creatinine (μmol/L)	70.15 [61.00,80.07]	88.96 [77.40,97.39]	<0.001
Uric acid (μmol/L)	381.46 [301.10,465.56]	449.37 [373.06,534.80]	<0.001
SaO_2 (%)	89.4 [86.6,92.5]	90.9 [88.7,93.9]	0.001
**Comorbidities**			
Thrombophilia	8 (5.0)	23 (12.1)	0.020
APS	12 (7.5)	14 (7.4)	0.963
CHD	6 (3.8)	17 (9.0)	0.05
DM	4 (2.5)	8 (4.2)	0.381
Hypertension	37 (23.1)	47 (24.7)	0.725
Cancer	3 (1.9)	1 (0.5)	0.336

Anemia	14 (8.8)	14 (7.4)	0.635
**Targeted drug at diagnosis**			0.528
No drugs	8 (5.0)	15 (7.9)	
Monotherapy	54 (33.8)	65 (34.2)	
Combination therapy	98 (61.3)	110 (57.9)	
**Main treatment modality**			0.038
Targeted drug	49 (30.6)	57 (30.0)	
PEA	46 (28.8)	77 (40.5)	
BPA	65 (40.6)	56 (29.5)	
**Anticoagulants**			0.043
Warfarin	55 (34.6)	86 (45.3)	
NOAC	104 (65.4)	104 (54.7)	
**Death**	22 (13.8)	23 (12.1)	0.647

Data are presented as mean±SD or n (%) or median [IQR].

CTEPH: chronic thromboembolic pulmonary hypertension; BMI: Body mass index ; VTE: Venous Thrombus Embolism; WHO FC: WHO function class; 6MWD: 6-minutes walking distance; PAP: pulmonary artery pressure; RAP: right atrial pressure; PAWP: pulmonary artery wedge pressure; PVR: pulmonary vascular resistance; CO: cardiac output; CI: cardiac index; SvO_2: mixed venous oxygen saturation; HGB: hemoglobin; WBC: white blood cell; PLT: platelet; NT-proBNP : N-terminal pro brain natriuretic peptide; ALT: alanine aminotransferase; AST: aspartic transaminase; TB: total bilirubin; DB: direct bilirubin; Sao_2: arterial oxygen saturation; APS: antiphospholipid syndrome; CHD: coronary heart disease; DM: Diabetes mellitus ; PEA: pulmonary endarterectomy; BPA: balloon pulmonary angioplasty; NOAC: novel oral anticoagulants.

Table S2. Univariate COX proportional hazards regression analysis of risk factors for mortality in female patients.

Variables	HR	95% CI	*P* value
**Characteristics**			
Age	1.00	0.97-1.03	0.831
BMI (kg/m^2)	0.91	0.80-1.04	0.156
Smoking	0.00	(0,inf)	0.993
Alcohol Drinking	—	—	—
History of VTE	0.61	0.26-1.42	0.255
Time from first symptom to diagnosis (months)	1.00	1.00-1.01	0.192
WHO FC III/IV	1.31	0.51-3.34	0.578
6MWD/20 (m)	0.92	0..86-0.99	0.018
**Hemodynamics**			
Systolic PAP (mmHg)	1.00	0.98-1.02	0.773
Diastolic PAP (mmHg)	1.01	0.96-1.06	0.743
mean PAP (mmHg)	1.01	0.98-1.04	0.612
mean RAP (mmHg)	1.04	0.96-1.13	0.364
PAWP (mmHg)	1.06	0.95-1.19	0.271

PVR (Wood units)	1.05	0.96-1.14	0.290
CO (L/min)	0.82	0.56-1.21	0.316
CI (L/(min.m^2)	0.82	0.41-1.64	0.565
Svo_2 (%)	0.94	0.90-0.98	0.006
**Laboratory tests**			
HGB (g/L)	0.98	0.97-1.00	0.053
PLT ($\times 10^9$)	1.00	0.99-1.00	0.431
NT-proBNP/500 (ng/ml)	1.07	1.01-1.13	0.017
D-dimer (μg/ml)	1.06	0.88-1.27	0.554
TB (μmol/L)	1.01	0.99-1.04	0.268
DB (μmol/L)	1.05	0.99-1.11	0.102
Creatinine (μmol/L)	1.01	1.00-1.03	0.119
Uric acid (μmol/L)	1.00	1.00-1.01	0.520
Sao_2 (%)	1.00	0.93-1.07	0.994
**Comorbidities**			
Thrombophilia	0.95	0.13-7.09	0.960
APS	0.93	0.12-7.04	0.946
CHD	3.36	0.78-14.57	0.105
DM	1.35	0.18-10.17	0.769
Hypertension	0.64	0.22-1.91	0.427
Cancer	9.34	1.13-76.90	0.038
Anemia	2.80	1.02-7.70	0.046
**Targeted drug**	1.10	0.54-2.27	0.791
**Main treatment modality**			
Targeted drug	Ref	Ref	
PEA	0.38	0.13-1.17	0.091
BPA	0.30	0.10-0.91	0.033
**Anticoagulants**			
Warfarin	Ref	Ref	
NOAC	1.57	0.63-3.91	0.333

BMI: Body mass index ; VTE: Venous Thrombus Embolism; WHO FC: WHO function class; 6MWD: 6-minutes walking distance; PAP: pulmonary artery pressure; RAP: right atrial pressure; PAWP: pulmonary artery wedge pressure; PVR: pulmonary vascular resistance; CO: cardiac output; CI: cardiac index; SvO_2: mixed venous oxygen saturation; HGB: hemoglobin; WBC: white blood cell; PLT: platelet; NT-proBNP : N-terminal pro brain natriuretic peptide; ALT: alanine aminotransferase; AST: aspartic transaminase; TB: total bilirubin; DB: direct bilirubin; Sao_2: arterial oxygen saturation; APS: antiphospholipid syndrome; CHD: coronary heart disease; DM: Diabetes mellitus ; PEA: pulmonary endarterectomy; BPA: balloon pulmonary angioplasty; NOAC: novel oral anticoagulant; Ref: reference.

Table S3. Clinical characteristics of female CTEPH Patients in each treatment group.

Variables	Targeted drug (n=49)	PEA (n=46)	BPA (n=65)	*P1*	*P2*	*P3*
**Characteristics**						
Age	58.0±12.9	47.8±11.8	57.2 14.1	<0.001	0.758	<0.001
BMI (kg/m^2)	24.2±3.1	23.0±3.6	23.6±3.1	0.095	0.333	0.353
Smoking	0 (0.0)	1 (2.2)	1 (1.5)	0.484	>0.999	>0.999
Alcohol Drinking	0 (0.0)	0 (0.0)	0 (0.0)	-	-	-
History of VTE	34 (69.4)	33 (71.7)	46 (70.8)	0.826	0.873	0.912
Time from first symptom to diagnosis (months)	27.3 [12.2,60.4]	11.6 [6.13,36.53]	33.9 [12.2,73.6]	0.007	0.473	0.001
WHO FC III/IV	32 (65.3)	30 (65.2)	46 (70.8)	0.993	0.535	0.535
6MWD (m)	357.0 [342.0,442.0]	408.0 [342.0,443.0]	370.0 [327.0,442.0]	0.201	0.818	0.115
**Hemodynamics**						
Systolic PAP (mmHg)	86.3±23.0	81.8±23.6	85.5±24.3	0.342	0.847	0.426
Diastolic PAP (mmHg)	31.0 [26.0,39.0]	29.0 [23.0,33.0]	31.0 [27.0,38.0]	0.159	0.932	0.099
mean PAP (mmHg)	52.0±13.3	48.6±13.2	50.9±12.6	0.211	0.648	0.351
mean RAP (mmHg)	7.0 [5.0,11.0]	6.0 [4.0 ,9.0]	7.0 [4.0,10.0]	0.099	0.776	0.189
PAWP (mmHg)	9.0 [8.0,11.0]	10.0 [8.0,11.0]	10.0 [8.0,12.0]	0.922	0.519	0.357
PVR (Wood units)	10.3 [8.1,13.3]	8.26 [6.33,10.53]	9.41 [6.71,12.41]	0.053	0.312	0.262
CO (L/min)	4.10 [3.67,4.60]	4.38 [4.07,5.13]	4.17 [3.47,5.13]	0.032	0.405	0.238
CI (L/(min.m^2)	2.41 [2.17,2.72]	2.55[2.28,3.06]	2.52 [2.09,2.96]	0.069	0.725	0.405
Svo_2 (%)	61.8 [57.7,65.1]	60.6 [56.3,65.5]	62.0 [55.9,67.5]	0.979	0.413	0.765
**Laboratory tests**						
HGB (g/L)	141.0[129.0,147.0]	141.0 [118.0,159.0]	145.0 [131.0,158.0]	0.829	0.279	0.563

WBC (×10[9])	5.63 [4.94,6.65]	5.73 [4.75,7.68]	5.78 [4.65,7.03]	0.663	0.891	0.664
PLT (×10[9])	192.0[162.0,258.0]	216.5 [171.0,273.0]	198.0 [155.0,244.0]	0.214	0.742	0.135
NT-proBNP (ng/ml)	1396.80 [560.30,2877.30]	1579.5 [310.8,2688.0]	1186.0 [227.0,2941.0]	0.704	0.520	0.728
D-dimer (μg/ml)	0.38 [0.20,0.80]	0.56 [0.26,0.93]	0.36 [0.17,0.75]	0.153	0.961	0.097
ALT (IU/L)	22.0 [14.0,35.0]	26.0 [13.0,41.0]	16.5 [12.0,25.5]	0.520	0.173	0.128
AST (IU/L)	21.0 [18.0,31.0]	26.0 [21.0,32.0]	16.5 [12.0,25.5]	0.053	0.229	0.348
ALP (IU/L)	67.0 [54.0,77.0]	79.0 [63.0,100.0]	65.0 [53.0,81.0]	0.004	0.898	0.006
Total bilirubin (μmol/L)	20.00 [14.30,29.60]	14.21 [10.75,20.19]	18.30 [13.21,28.90]	0.009	0.375	0.072
Direct bilirubin (μmol/L)	3.60 [2.90,7.20]	3.10 [2.40,4.90]	4.58 [3.25,8.28]	0.115	0.160	0.007
Creatinine (μmol/L)	66.89 [55.90,73.66]	69.00 [60.31,80.07]	76.0 [66.8,84.0]	0.249	<0.001	0.032
Uric acid (μmol/L)	364.10 [284.7,446.44]	375.63 [286.64,459.51]	400.05 [337.50,467.28]	0.719	0.112	0.371
Sao_2 (%)	89.4 [87.3,92.4]	90.0 [86.7,93.1]	89.3 [85.9,91.8]	0.718	0.413	0.302
**Comorbidities**						
Thrombophilia	1 (2.0)	4 (8.7)	3 (4.6)	0.195	0.633	0.446
APS	3 (6.1)	4 (8.7)	5 (7.7)	0.709	>0.999	>0.999
Coronary disease	4 (8.2)	0 (0.0)	2 (3.1)	0.118	0.400	0.510
Diabetes mellitus	2 (4.1)	0 (0.0)	2 (3.1)	0.495	>0.999	0.510
Hypertension	16 (32.7)	5 (10.9)	16 (24.6)	0.011	0.344	0.069
Gastrointestinal disease	2 (4.1)	1 (2.2)	7 (10.8)	>0.999	0.296	0.137
Chronic kidney disease	0 (0.0)	0 (0.0)	1 (1.5)	-	>0.999	>0.999
Cancer	2 (4.1)	0 (0.0)	1 (1.5)	0.495	0.576	>0.999

Hyperhomocysteinemia	11 (22.5)	4 (8.7)	36 (55.4)	0.066	<0.001	<0.001
Anemia	4 (8.2)	5 (10.9)	5 (7.7)	0.735	>0.999	0.739
**Targeted drug at diagnosis**				0.004	<0.001	0.044
No drugs	0 (0.0)	69 (13.0)	2 (3.1)			
Monotherapy	27 (55.1)	14 (30.4)	13 (20.0)			
Combination therapy	22 (44.9)	26 (56.5)	50 (76.9)			
**Anticoagulants**				0.847	<0.001	<0.001
Warfarin	22 (45.8)	22 (47.8)	11 (16.9)			
NOAC	26 (54.2)	24 (52.2)	54 (83.1)			
**Combination Medicine**						
Antiplatelet	3 (6.1)	1 (2.2)	4 (6.2)	0.618	>0.999	0.401
NASID	1 (2.0)	2 (4.4)	1 (1.5)	0.609	>0.999	0.569
Steroidhormones	1 (2.0)	5 (10.9)	4 (6.2)	0.104	0.389	0.485
CCB	1 (2.0)	0 (0.0)	1 (1.5)	>0.999	>0.999	>0.999
Diuretic	41 (83.7)	41 (89.1)	61 (93.9)	0.439	0.080	>0.999
Digoxin	26 (53.1)	28 (60.9)	41 (63.1)	0.443	0.282	0.813
IVC filter	4 (8.2)	11 (23.9)	4 (6.2)	0.035	0.724	0.007

Data are presented as mean±SD or n (%) or median [IQR]. CTEPH: chronic thromboembolic pulmonary hypertension; BMI: Body mass index ; VTE: Venous Thrombus Embolism; WHO FC: WHO function class; 6MWD: 6-minutes walking distance; PAP: pulmonary artery pressure; RAP: right atrial pressure; PAWP: pulmonary artery wedge pressure; PVR: pulmonary vascular resistance; CO: cardiac output; CI: cardiac index; SvO_2: mixed venous oxygen saturation; HGB: hemoglobin; WBC: white blood cell; PLT: platelet; NT-proBNP : N-terminal pro brain natriuretic peptide; ALT: alanine aminotransferase; AST: aspartic transaminase; TB: total bilirubin; DB: direct bilirubin; Sao_2: arterial oxygen saturation; APS: antiphospholipid syndrome; CHD: coronary heart disease; DM: Diabetes mellitus ; PEA: pulmonary endarterectomy; BPA: balloon pulmonary angioplasty; NOAC: novel oral anticoagulants. *P1*: the *P* value between patients receiving targeted drug along and patients receiving PEA; *P2*: the *P* value between patients receiving targeted drug along and patients receiving BPA; *P3*: the *P* value between patients receiving PEA and patients receiving BPA. *P* <0.017 (Bonferroni correction) was consider significance.

Table S4. The results of sensitivity analysis.

Variables	HR	95% CI	*P* value
Age	1.00	0.96-1.04	0.997
BMI (kg/m^2)	0.92	0.78-1.09	0.357
6MWD/20 (m)	0.96	0.89-1.04	0.310
Svo_2 (%)	0.98	0.92-1.05	0.546
NT-proBNP/500 (ng/ml)	1.09	1.00-1.19	0.050
Cancer	4.97	0.48-51.76	0.180
Anemia	4.96	1.33-18.40	0.017
Time from first symptom to diagnosis (months)	1.00	0.99-1.01	0.689
Anticoagulants	1.67	0.61-4.57	0.317
**Main treatment modality**			
Targeted drug	Ref	Ref	-
PEA	0.25	0.06-0.99	0.049
BPA	0.13	0.03-0.60	0.009

Multivariate Cox analysis after adjusting the confounders (age and BMI) and survival-related factors. BMI: Body mass index; 6MWD/20: 6-min walk distance, per 20m increase in distance; SvO2: mixed venous oxygen saturation; NT-proBNP/500: N-terminal pro-brain natriuretic peptide, per 500 ng/ml increase in concentration; PEA: pulmonary endarterectomy; BPA: balloon pulmonary angioplasty.
